# Enteroclysis and Diarrheas

**Published:** 1903-08

**Authors:** 


### ABSTRACTS.

### Enteroclysis and Diarrheas.

Dr. Robert Coleman Kemp *(Manual on Enteroclysis, Hypodermoclysis, and Infusion)*, gives an introductory chapter by D . William H. Thomson, from which the following is an extract:

Lavage of the invisible cavities of the body in inflammatory states of their lining mucous membrane has justly taken rank among the most effective of modern remedial measures. In the case of the rectum and lower bowel, however, the greatest number of advantageous results follow from this procedure, not only by improvement in local conditions, but still more by effects obtainable through certain physiological relations upon the general circulation, as well as others secured through important nervous associations with contiguous organs. . . . Irrigation with plain hot water, or with normal saline solution, had proved very distressing, preventing sleep and thereby seriously impairing the general constitutional state, and against this complication I have found no one measure so effective as irrigation after every movement of the bowels. The addition to the water of such an antiseptic and local anodyne as five to ten drops of oleum menthol is also useful. In both acute and chronic colitis, especially the latter, I have often found medicinal remedies fail to complete the cure until they were supplemented by the local effects of this lavage.

In the treatment of cholera infantum, in chronic colitis and various dysenteric conditions, in typhoid fever and in gastro-intestinal affections of a catarrhal nature, washing out the lower bowel has become a well-recognised therapeutic measure, and for this purpose there is no better remedy than glycothymoline. Its action is to deplete inflammatory engorgements by exosmosis, increasing capillary circulation and maintaining aseptic cleanliness. When diluted to a 25 per cent. solution, glycothymoline has a saline strength and alkalinity similar to that of blood. It was conceived with the natural constituents of blood and secretions of the mucous membranes in full view. A local application to be successful, must harmonise with the natural fluids of the tissues to be treated. It readily dissolves accumulated mucus, detaching mucous crusts and necrosed tissue. The advantages of glycothymoline in gastrointestinal inflammations seem to consist in its being a nonirritant, antiseptic, and antifermentive remedy witnout interfering with the normal action of gastric ferments. Its local sedative, anodyne and healing properties, and its power as a nondepressant antipyretic; its depletive action on the congested gastrointestinal mucous membrane contribute in no small degree to the healing process which follows its administration.

Bearing upon this subject, the following cases occurring in the practice of Dr. Willis E. Cummings, of Brooklyn, N. Y., are interesting:

Mary A., 12 years old, gave a history of dysenteric discharges, extending over a period of two weeks. The excreta were very offensive, almost typhoid in character. My usual method of treatment in these cases during the past two years has been to prescribe either liquor bismuth or chalk mixture in combination with a glycothymoline base, but on account of the urgency of the symptoms in this case I flushed out the bowel with glycothymoline, one ounce to the pint of warm water. The effect of this treatment was most marked. The diarrhea was checked within six hours, the pain was less severe and the temperature fell.

Mrs. C., 52 years old, was suffering from a chronic diarrhea of ten years' standing, which gave every evidence of being of a tubercular nature. The usual remedies I had been giving produced no lasting effect. Having found glycothymoline very advantageous in the ordinary summer complaints of children, I made use of it in this case, flushing out the bowel about three times weekly, and giving two drams of the same remedy in half a glass of warm water every four hours during the day, by the mouth. The condition of the patient improved almost immediately, the tenderness and tympanitis disappeared, and in two weeks the patient presented apparently a normal bowel, and steadily gained in weight. This extremely satisfactory result I have decided was due largely to the correction of the fermentative processes, caused by improper secretion of the glands, restoring in a measure the healthful action of the same.

The following prescriptions and methods have been used for sometime, and may be regarded as standard:

Liquor bismuth and glycothymoline, of each, two ounces. Dose, a teaspoonful as often as may be required.

Glycothymoline may be combined with bismuth, tinct. opii camph; tinct. opii; mistura creta; syrup rhei aromatic, and similar medicines.

Administered internally, glycothymoline acts as a carminative, internal antiseptic, alterative, stimulant and antacid, and meets many of the requirements of the physician during the summer months.

Glycothymoline diluted, one ounce to the quart of water, used as a sponge bath, stimulates the skin secretions.

As an enema, glycothymoline, one ounce to the pint, will be found most valuable.

